# Fabrication of polyhedral Cu–Zn oxide nanoparticles by dealloying and anodic oxidation of German silver alloy for photoelectrochemical water splitting

**DOI:** 10.1038/s41598-022-21127-1

**Published:** 2022-10-06

**Authors:** Nour Bahnasawy, Abdussalam M. Elbanna, Mohamed Ramadan, Nageh K. Allam

**Affiliations:** Energy Materials Laboratory, School of Sciences and Engineering, The American University in Cairo, New Cairo, 11835 Egypt

**Keywords:** Devices for energy harvesting, Hydrogen fuel

## Abstract

A significant effort has been dedicated to the synthesis of Cu–Zn oxide nanoparticles as a robust photocathode material for photoelectrochemical water splitting. Cu–Zn oxide nanoparticles were formed by controlled anodization of German silver (Cu–Zn–Ni) alloy in an aqueous electrolyte. Scanning electron microscopy (SEM) demonstrates the dependence of the obtained nanostructures on the anodization time. The X-ray diffraction (XRD) patterns showed the formation of copper oxide (CuO) and zinc oxide (ZnO) nanoparticles with good stability. This was also confirmed by the compositional X-ray photoelectron spectroscopy (XPS) analysis. The obtained polyhedral nanoparticles showed high optical activity with adequate bandgap energy. These optimized nanoparticles achieved boosted photocurrent of − 0.55 mA/cm^2^ at − 0.6 V vs. SCE under AM 1.5 illumination, confirming the role of the optimized dealloying and thermal treatment in tuning the photoelectrochemical performance of the material.

## Introduction

Extensive research has been conducted to improve the performance of photoelectrochemical water splitting systems^1–3^. The scarcity of low-cost, earth-abundant, photoactive, and stable photoelectrodes is the challenge in realizing the commercialization of such systems^4^. To this end, earth-abundant metal oxides can be utilized as effective photoelectrodes to attain this purpose^5–7^. Among several metal oxides, copper oxides are considered a class of the best metal oxide semiconductors due to their high absorption over a wide range of the solar spectrum and being cost-effective, non-toxic, and abundant in nature^8–12^. Despite their excellent electronic transport properties, absorption coefficient, and direct bandgap^9–13^, copper oxides are unstable, hindering their wide use in aqueous electrolyte-based systems. Hence, more efforts have been devoted to improving their stability, such as coating, doping with several metals and nonmetals^14^, annealing treatments^15^, and nanostructuring^16^. Still, among those used protocols are severe artifacts^17^. On the contrary, ZnO has high stability but a wide bandgap^18–20^, which restricts its absorption to the UV region of the light spectrum. Several efforts were exerted to improve its optical activity, including nanostructuring^21^, decoration^22^, and doping^20,21^, but with limited success.

Moreover, nanoscale materials have a plethora of merits over bulk materials, including the high surface area, enhanced physical properties, and tuned electronic properties^23^. The more surface area, the more active sites are available for reactions to occur. Moreover, the very small size (lower than 10 nm) results in quantum confinement effects that improve the charge carriers separation and transport. In addition, these nanomaterials can be produced in different morphologies, such as tubes, rods, flowers, spheres, and many more, enabling tuned characteristics^24^.

Unfortunately, the overall water splitting efficiency is hindered by the sluggish kinetics of the oxygen evolution reaction (OER) at the anode that involves a complex, four-electron/proton transfer process^25^. Over the last few years, RuO_2_ and IrO_2_-based materials are considered the benchmark electrocatalysts for OER due to their high electrocatalytic efficiencies and outstanding carrier mobilities. On the other hand, noble platinum (Pt)-based materials are the most efficient electrocatalysts and the benchmarks for the hydrogen evolution reaction (HER) due to lower Tafel slopes and overpotentials. However, the higher cost of those materials limits their widespread applications^26,27^. Hence, exploring alternative low-cost and high-performance water-splitting catalysts is neccessary. As a result, many efforts have been devoted to tune the cost effective transition metal-based materials for use as non-noble metallic catalysts^28^. Specifically, multi-transition metallic alloy electrocatalysts have stimulated great interest in water splitting applications due to their intrinsic electrocatalytic activity and ease of manipulating their electronic structure^29^.

To this end, the combination of CuO and ZnO nanostructures has been widely studied in photocatalytic systems with the aim of integrating the complimentary properties of each oxide^30–32^. The CuO–ZnO heterostructures were prepared by a plethora of methods, including electrochemical deposition and radiofrequency magnetron sputtering. However, these techniques require a long processing time, high temperatures, and are costly. Hence, it is of great importance to identify a more straightforward process to fabricate CuO–ZnO heterostructured nanomaterials. To this end, electrochemical anodization is a surface modification technique that is extensively used to create vast nanostructures of several metals and alloys for different applications^7,16,33–35^.

Herein, we report on the optimized fabrication of CuO–ZnO heterostructures with controlled morphology via simple anodization of German silver alloy (Cu–Zn–Ni) followed by air annealing. The produced nanoparticles were investigated as photocathodes to split water photoelectrochemically. It is worth noting that this is the first study on the fabrication of photocathode nanocatalysts via the anodization of German silver alloy and their application in water splitting systems. Note that a current density of CuO of − 0.48 mA/cm^2^ at 0.473 V vs. RHE was reported, which is lower than ours even at much lower potential^36,37^.

## Experimental section

Before anodization, the German silver alloy (65% Cu–22% Zn–13% Ni) was mechanically polished with grinding paper to remove the native oxide. Furthermore, three samples were immersed in dilute HCl, then washed with deionized water. After that, the samples were ultrasonicated in ethanol, water, and acetone for 10 min each. The samples were anodized at room temperature under a constant voltage of 4 V for 2, 5, and 8 min in 0.1 M APS (1 mL), 2.5 M NaOH (1 mL), and 48 mL of deionized water. The distance between the two electrodes is fixed at 1.5 cm. The anodized samples were annealed in air at 350 °C for 1 h with a ramping rate of 5 °C/min. The morphology and the composition of the films are illustrated using a Zeiss SEM Ultra 60 FESEM machine with an accelerating voltage of 4 kV. The crystal structure of the annealed sample was determined using a PANalytical X-pert Pro PW3040 MPD X-ray diffractometer via monochromatic radiation (Cu-Kα, λ = 0.15406 nm, 50 mA, 40 kV) in the range of 5°–80° with a glancing angle of 0.5°. The optical characterization of the fabricated photocathode was performed using a Shimadzu UV–Vis diffuse reflectance spectrometer, and their optical band gap energy was calculated using a Tauc plot. The photoelectrochemical analysis was performed in 1.0 M Na_2_SO_4_ in a three-electrode cell with an SCE electrode as a reference electrode, platinum as a counter electrode, and the photocathode as the working electrode using Bio-Logic SP 200 potentiostat using a 300 W Ozone-free Xenon lamp under 100 mW/cm^2^ illumination equipped with AM 1.5G filter.

## Results and discussion

**Figure 1:**
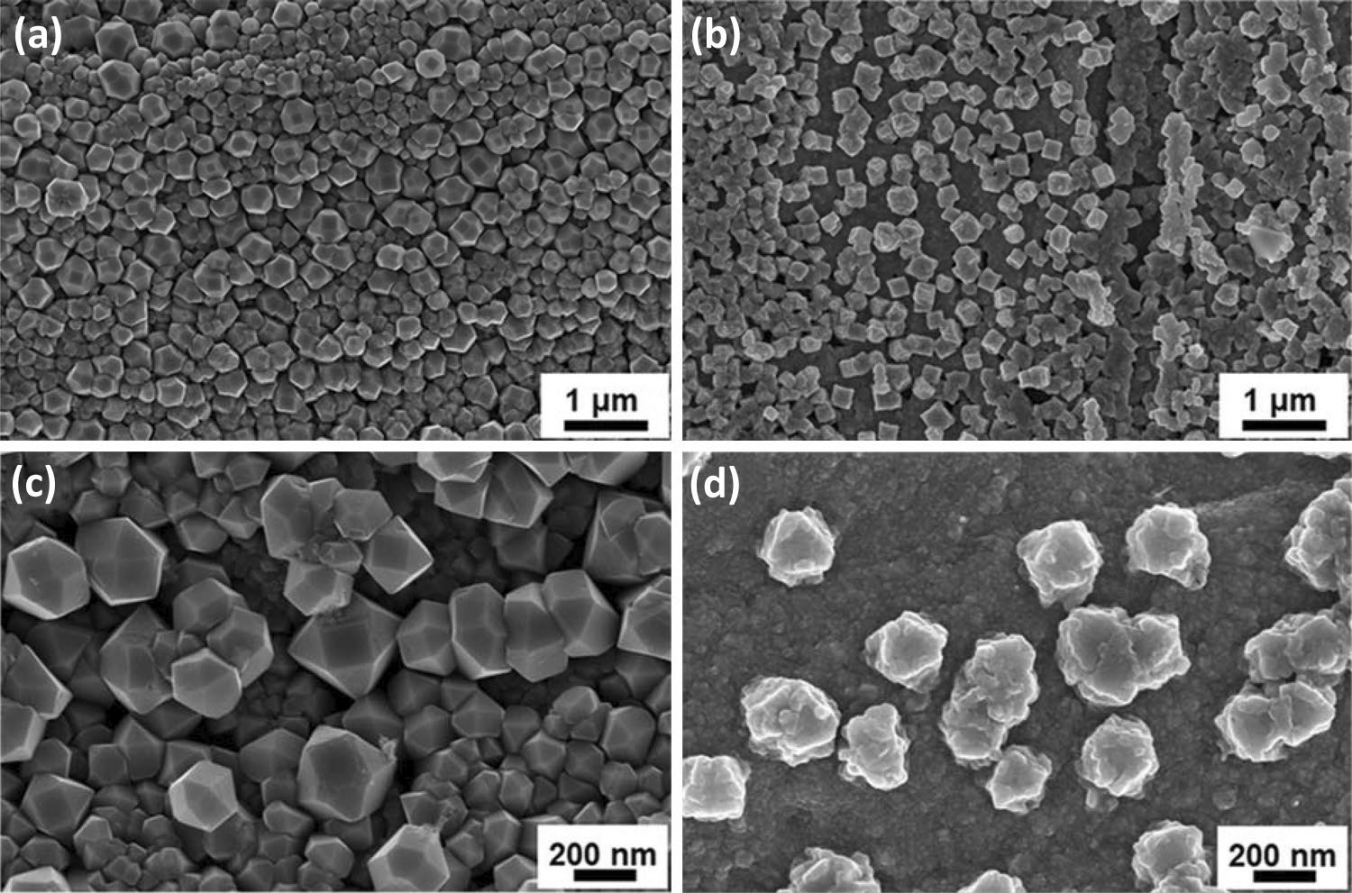
FESEM images of the fabricated photocathodes (**a,c**) before and (**b**,**d**) after annealing at 350 °C in the air for 1 h.

The morphology of the fabricated samples before and after annealing was inspected using FESEM, as presented in Fig. 1. The surface of the as-anodized sample is entirely covered with homogeneous polyhedral nanoparticles, Fig. 1a,c. The polyhedral nanoparticles were achieved upon anodization of the German silver alloy in the aqueous electrolyte containing 0.1 M ammonium persulfate (APS) and 2.5 M NaOH at 4 V for different times. The oxidation of the alloy formed oxide nanoparticles, and the process was accelerated by using the APS oxidant. We believe the oxidation process in sodium hydroxide occurs in a few steps. First, lower metal oxide (e.g., Cu_2_O) is formed, then oxidized to the higher oxide (e.g., CuO), and finally, metal hydroxide (e.g., Cu(OH)_2_) covers the outer layer of the oxide structures^38,39^. Moreover, the present persulfate ions in the electrolyte cause pitting of the formed oxide layer leading to the formation of the observed polyhedral nanoparticles^40^ The FESEM image of the as-anodized samples reveals a uniform distribution and good interconnectivity of the formed oxide polyhedral nanoparticles. The as-anodized samples were calcined at 350 °C for 1 h in ambient air to dehydrate and complete the conversion of the hydroxide into the corresponding oxide (Fig. 1b)^41^. However, upon annealing, some of the polyhedral nanoparticles tend to agglomerate (Fig. 1d) due to the difference in the thermal expansion coefficient between the film and the substrate^42,43^.

**Figure 2:**
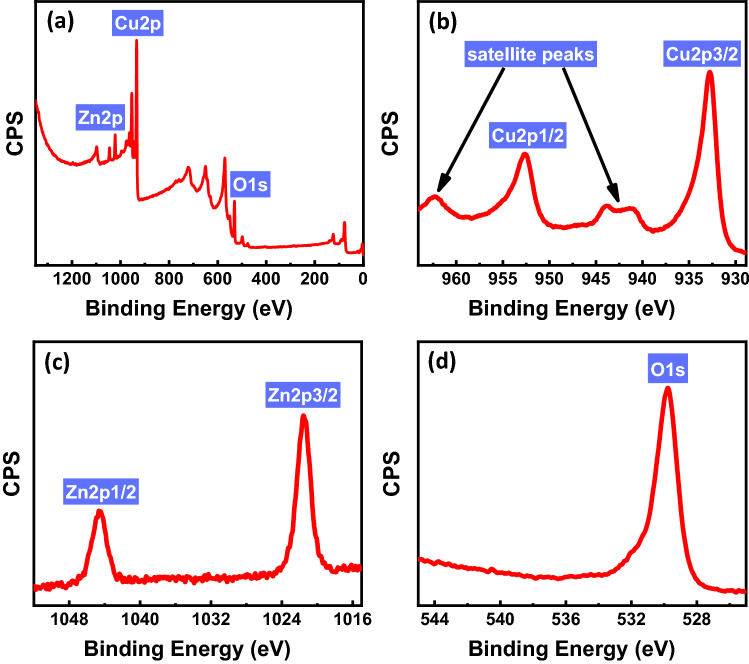
(**a**) XPS survey spectra, high-resolution XPS scans of (**b**) Cu 2p, (**c**) Zn 2p, and (**d**) O 1 s for the fabricated photocathode annealed at 350 °C in the air for 1 h.

To get a better understanding of the composition of the fabricated photocathodes, an XPS analysis was conducted. Figure 2a illustrates the survey spectra of the annealed photocathode, which demonstrate the existence of Cu, Zn, and O peaks. Figure 2b,c show the spectra of Cu 2p and Zn 2p, respectively. The Cu 2p spectrum exhibits two peaks at 932.69 eV and 952.54 eV, attributing to Cu 2p_3/2_ and Cu 2p_1/2_, respectively. The satellite peaks at 941.21 eV, 943.77 eV, and 962.02 eV are related to the bivalent energy state of copper, characteristic of Cu^2+^^10,12,44,45^. The peaks at 1021.54 and 1044.62 eV correspond to the Zn 2p_3/2_ and Zn 2p_1/2_, respectively. The observed difference between these binding energies was found to be 23.08 eV, confirming the Zn^2+^ oxidation state^46–49^. The peak at 529.75 eV, Fig. 2d, is characteristic of lattice oxygen in metal oxides. Thus, the XPS results support the formation of CuO and ZnO.

**Figure 3:**
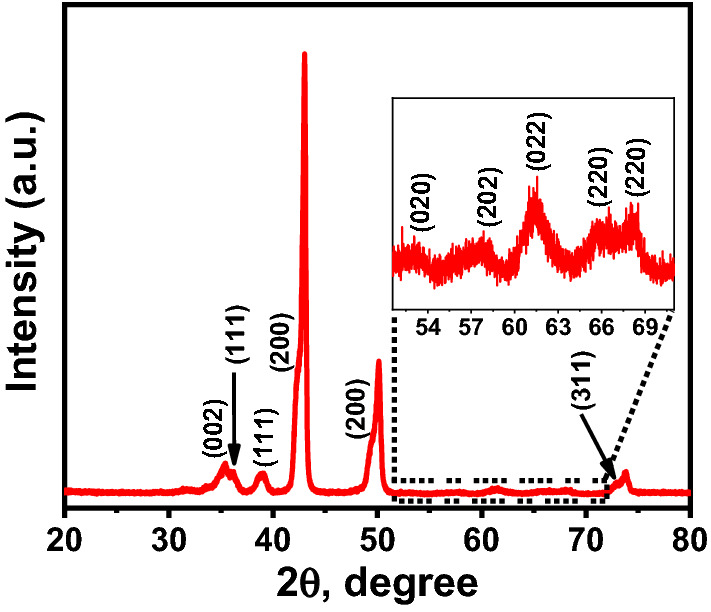
XRD pattern of the fabricated photocathode annealed at 350 °C in the air for 1 h.

Figure 3 shows the XRD pattern of the fabricated photocathode. The peaks observed at 35.43°, 38.85°, 53.30°, 58.03°, 65.61°, 68.16°, and 72.69° can be ascribed to the (002), (111), (020), (202), (022), (220), and (311) planes of monoclinic CuO (JCPDS# 00-041-0254)^50–52^. Moreover, the peaks appeared at 36.23°, 42.12°, 49.39°, and 61.44° can be assigned to the (111), (200), (200), and (220) planes of cubic ZnO (JCPDS# 01-077-0191) (JCPDS# 01-078-46100)^47,53^. The peaks that appeared at 43.09°, 50.15°, and 73.86° can be related to the substrate^54^. Thus, the XRD spectra indicate that the sample's surface is covered by a thin film of mixed CuO and ZnO. The average crystallite sizes (*D*) of CuO and ZnO nanoparticles were calculated using the Scherrer formula:

1 $$D=\frac{K\lambda }{\beta \mathrm{cos}\theta }$$

where *θ* is the Bragg diffraction angle, λ is the X-ray wavelength, and β is the full width at half maximum of the XRD peak existing at a diffraction angle θ. The average crystallite sizes for CuO and ZnO nanoparticles were calculated to be 34.64 and 32.52 nm, respectively^55,56^.

**Figure 4:**
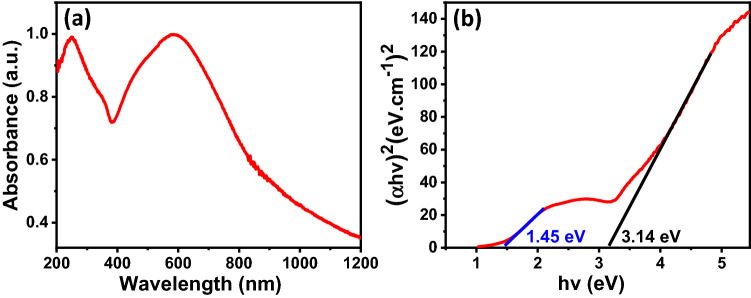
(**a**) UV–Vis absorption spectra and (**b**) Tauc plot of the fabricated photocathode annealed at 350 °C for 1 h in air.

The optical activity of the fabricated photocathode was tested using a UV–Vis spectrometer. Figure 4a shows the absorption spectrum of the fabricated photocathode. Two prominent absorption bands were observed at 855 nm and 394 nm, ascribed to the characteristic absorption of CuO and ZnO, respectively^57,58^. To estimate the bandgap energy of the material, Tauc plot was constructed according to Eq. (2):

2 $$\left( {\alpha {\text{hv}}} \right)^{{\text{n}}} {\text{ = A}}\left( {hv{-}{\text{ E}}_{{\text{g}}} } \right)$$

where *α* is the absorption coefficient obtained from UV–Vis spectra, *hv* is the photon's energy, *A* is a constant, *E*_*g*_ is the optical bandgap energy, and *n* depends on the nature of the transition in a semiconductor. As shown in Fig. 4b, straight lines are determined when (*αhv*)^2^ is plotted *versus* photon energy (*hv*), indicating that the absorption is due to a direct transition (*n* = 2) for both CuO^59,60^ and ZnO^61,62^. The bandgap energies of CuO and ZnO were found to be 1.45 eV and 3.14 eV, respectively. These values are in good agreement with those previously reported^63–66^.

**Figure 5:**
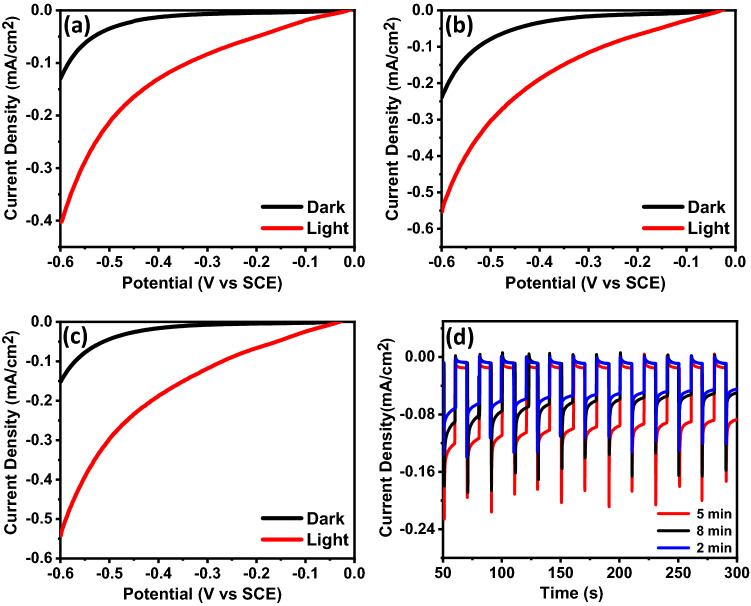
*J–V* and *J–t* curves of annealed Cu–Zn oxide photocathodes that anodized for (**a**) 2 min, (**b**) 5 min, (**c**) 8 min, and (**d**) the transient photocurrent (J–t).

To photoactivity of the fabricated photocathodes was evaluated in a three-electrode photoelectrochemical cell. Figure 5 shows the linear sweep voltammetry (LSV) of the photocathode in the dark and under illumination. An observed increase in the current density was shown for all samples when exposed to light, as shown in Fig. 5a–c. Eventually, these photo-induced cathodic currents indicate the p-type behavior of copper oxide nanoparticles^67^. The sample anodized for 2 min showed the lowest photocurrent density compared to those counterpart samples prepared by anodization at longer times. This can be ascribed to the lack of active sites that are exposed to light and may indicate the incomplete nanostructuring of the whole surface of the substrate at shorter anodization time. However, at 8 min of anodization, the covering on the surface of polyhedral nanoparticles will restrain the entry of sunlight^68,69^. The sample anodized for 5 min showed an increased photocurrent of − 0.55 mA/cm^2^ at − 0.6 V vs. SCE, which can be ascribed to the increased light absorption. Although the sample anodized for 8 min showed approximately the same photocurrent as the sample anodized for 5 min, the dark current was lower.

To examine the photostability of the fabricated photocathodes, the *J–t* curves of the fabricated photocathodes were recorded at a constant potential of − 0.4 V vs. SCE for 300 s under dark and illumination, as shown in Fig. 5d. The sample anodized for 5 min showed the highest photocurrent density, indicating good charge separation and collection compared to other samples. Besides, the samples anodized for 2 and 8 min showed nearly similar photocurrent density. Note that the observed photocurrent decay occurs as a result of the photoreduction of CuO under illumination to form Cu_2_O and, finally, copper. The samples anodized for 2 min and 8 min showed decay in photocurrent density of 45% and 50% after 300 s, respectively.

On the other hand, the sample anodized for 5 min retrieved better stability with 68% photocurrent retention after 300 s. Hence, the sample anodized for 5 min is considered the best electrode among the other fabricated samples^57^. The photocurrent density of our fabricated electrodes is much higher than those reported for other trimetallic systems for photoelectrochemical water splitting^7,70–74^.

## Conclusion

In summary, a simple synthesis method of polyhedral Cu–Zn oxide nanoparticles via anodization followed by annealing treatment is demonstrated. The XPS analysis indicated the formation of Cu–Zn-O. Moreover, the XRD analysis confirmed the existence of both CuO and ZnO. The UV–Vis absorption spectra showed the material to have two absorption band edges with corresponding bandgap energies of 1.45 eV and 3.14 eV, in agreement with the materials characterization data. The electrodes showed a photocurrent of − 0.55 mA/cm^2^ at − 0.6 V vs. SCE under AM 1.5 illumination, which is much higher than those reported for other trimetallic systems. Accordingly, this study introduces avenues to design and fabricate stable and photoactive electrodes for photoelectrochemical hydrogen production.
